# The impact of COVID-19 on disease epidemiology, family dynamics, and social justice in Minnesota: All that you cannot see

**DOI:** 10.1017/cts.2022.422

**Published:** 2022-06-27

**Authors:** Mark R. Schleiss, Bruce Blazar, Emily P. Chapman, Gretchen J. Cutler, Diana B. Cutts, Milton Mickey Eder, Shengxu Li, Susan M. Mason, Brianna M. Bretscher, Joseph P. Neglia, Peter B. Scal, Stuart S. Winter

**Affiliations:** 1 University of Minnesota Medical School, Minneapolis, MN, USA; 2 Children’s Hospitals and Clinics of Minnesota, Minneapolis, MN, USA; 3 Hennepin County Medical Center, Minneapolis, MN, USA; 4 University of Minnesota School of Public Health, Minneapolis, MN, USA

**Keywords:** Community networks, community-based participatory research, partnerships, COVID-19

## Abstract

**Objective:**

The COVID-19 pandemic presented a challenge to established seed grant funding mechanisms aimed at fostering collaboration in child health research between investigators at the University of Minnesota (UMN) and Children’s Hospitals and Clinics of Minnesota (Children’s MN). We created a “rapid response,” small grant program to catalyze collaborations in child health COVID-19 research. In this paper, we describe the projects funded by this mechanism and metrics of their success.

**Methods:**

Using seed funds from the UMN Clinical and Translational Science Institute, the UMN Medical School Department of Pediatrics, and the Children’s Minnesota Research Institute, a rapid response request for applications (RFAs) was issued based on the stipulations that the proposal had to: 1) consist of a clear, synergistic partnership between co-PIs from the academic and community settings; and 2) that the proposal addressed an area of knowledge deficit relevant to child health engendered by the COVID-19 pandemic.

**Results:**

Grant applications submitted in response to this RFA segregated into three categories: family fragility and disruption exacerbated by COVID-19; knowledge gaps about COVID-19 disease in children; and optimizing pediatric care in the setting of COVID-19 pandemic restrictions. A series of virtual workshops presented research results to the pediatric community. Several manuscripts and extramural funding awards underscored the success of the program.

**Conclusions:**

A “rapid response” seed funding mechanism enabled nascent academic-community research partnerships during the COVID-19 pandemic. In the context of the rapidly evolving landscape of COVID-19, flexible seed grant programs can be useful in addressing unmet needs in pediatric health.

## Introduction

Developing and nurturing partnerships between academic biomedical training programs and community-based medical centers is one of the overarching goals of the Clinical and Translational Science Award (CTSA) funding mechanism [1]. Beginning in 2012, the University of Minnesota (UMN) Clinical and Translational Science Institute (CTSI), along with the UMN Medical School Department of Pediatrics, has engaged in a research partnership with Children’s Hospitals and Clinics of Minnesota (Children’s MN), a free-standing affiliated community hospital system. The CTSI has child health research representation in the broadest sense, including partnerships with other Academic Health Center (AHC) colleges (UMN Schools of Public Health, Dentistry, Pharmacy, Veterinary Medicine, and Nursing), as well as partnerships with Children’s MN, a major provider of pediatric care in the community, and the Hennepin County Medical Center (HCMC), another care system providing both inpatient and outpatient pediatric services. These longstanding partnerships have focused on activities that include dinner programs highlighting community research initiatives shared between Children’s MN and the UMN; the issuance of an annual request for applications (RFA) for a Collaborative Research Award, funded through equal contributions by the three partners (the UMN CTSI, the UMN Department of Pediatrics, and Children’s MN); and the formation of a Child Health Advisory Board, comprised of members representing the UMN AHC, the HCMC Department of Pediatrics, and Children’s MN. Collaborative activities have included the preparation of the grant RFA, a NIH study section-style review of funding applications, administration of the grant award, and participation in strategic planning sessions.

The COVID-19 pandemic generated great challenges in the conduct of clinical and translational research. Indeed, in the beginning of the pandemic, it is estimated that some 80% of non-COVID related clinical trials were stopped [2]. COVID-19-related challenges included, but were by no means limited to, challenges in recruitment during periods of clinic “lock-down”; heightened awareness of numerous existing vulnerabilities that contribute to health disparities; challenges for institutional review boards (IRBs) in coordinating consent and enrollment procedures; concerns about safety, particularly related to biorepositories; disruption to research supply chains for project-related materials, equipment, and supplies; and difficulties in research administration [3–7]. In response to the pandemic, and recognizing the challenges both in sustaining existing clinical research studies and in initiating new, novel studies to address the impact of the SARS-CoV-2 coronavirus on child health, the UMN and Children’s MN fundamentally changed the collaborative grant program. Prior to the pandemic, RFAs were issued with the goal of funding one or two large ($150,000–$200,000) projects during each funding cycle. The RFAs have always focused on research, with a pre-requisite that the applications be driven by a demonstrable research partnership between investigators at the respective institutions. High-priority areas emphasized in the RFAs in the past included childhood mental health/behavioral health/substance abuse; health disparities; community population health (including rural health); and social determinants of health (homelessness; food insecurity; transportation; language barrier; violence). Examples of funded projects in past grant cycles included food insecurity, pediatric obesity, the impact of structural racism on child health, and diabetes education in Minnesota’s Somali-American community. Although these collaborations have been highly successful, in light of the COVID-19 pandemic, we perceived an urgent need to shift this collaborative funding mechanism to a rapid response mechanism to facilitate community-university collaborations addressing pediatric-specific aspects of this emerging public health crisis.

## Materials and Methods

### Release of RFA

The onset of the COVID-19 pandemic in early 2020 precipitated considerable disruption of programmatic planning of collaborative research priorities between the UMN CTSI and Children’s MN. Consideration was therefore given to utilizing the power of this partnership to repurpose the previous collaborative research proposal RFA to focus on seed grants aimed at facilitating rapid responses to the COVID-19 pandemic. Toward this goal, an RFA was released on April 7, 2020 representing the established partnership between the UMN CTSI, the UMN Medical School Department of Pediatrics, and Children’s MN. The goals were to fund nine seed grants of $10,000 each (based on available funds) that would address knowledge gaps in COVID-19 disease as it related to children’s health, and to continue to facilitate the formation of nascent partnerships that would enable collaboration between our respective institutions.

### RFA Deliverables

The RFA was released to the child health community in Minnesota, including individuals holding faculty positions in the UMN AHC as well as community partners in the Children’s MN network. Table 1 summarizes key features of the RFA. Although the RFA was not restrictive (any topic was welcome, as long as it was related to the impact of the COVID-19 pandemic on children’s health and had both a UMN and Children’s MN partner), three thematic areas were emphasized (Table 1).

**Table 1.** Potential areas of emphasis proposed in child health COVID “rapid response” collaborative grant request for applications (RFA)

Areas of particular interest emphasized for collaborative child health COVID-19 RFA	
Targeted area	Examples
Family fragility and disruption	Unemployment/economic burden Loss of educational opportunities Poverty Impaired access to well-child care Homelessness Food insecurity Loss of connection to a family’s faith community
Knowledge gaps about COVID-19 in children	Biology and epidemiology of COVID-19 infection in children Sequelae of COVID including MIS-C Enhancing understanding of the virologic, epidemiologic, and clinical consequences of COVID-19 including differences in disease between children and adults
Optimizing pediatric care in the setting of the COVID-19 pandemic	Telemedicine and computer-based approaches to health care Impact of COVID-19 on routine care, immunizations, access to social services Impact of school closures, social isolation on childhood mental health

#### Family Fragility and Disruption

The social, psychological, and societal burdens facing families – in addition to the medical consequences of COVID-19 – were identified as issues that were considered to be likely to contribute to social upheaval in the context of the COVID-19 pandemic. Familial disruptions, unemployment, loss of educational opportunities, poverty, impaired access to well-child care, homelessness, food insecurity, and a loss of connection to a family’s faith community were examples of areas of impact that COVID-19 might have on child health and were cited as areas of high priority for building research partnerships.

#### Knowledge Gaps about COVID-19 in Children

The RFA noted that a popularly stated view had emerged in the lay community and in social media circles that infants and children were much less affected medically by COVID-19 than were adults. However, the RFA emphasized that this was not strongly evidence-based and that more data were needed. The RFA sought applications that addressed unmet needs in learning about the biology and epidemiology of COVID-19 infection in children. Projects aimed at improving understanding of virologic, epidemiologic, and clinical consequences of COVID-19 infection were particularly welcome.

#### Optimizing Pediatric Care in the Setting of the COVID-19 Pandemic

It was noted that no clear consensus existed on optimal approaches to inpatient and outpatient care early in the course of the pandemic. It was also noted that in settings where diagnostic tests and personal protective equipment were lacking, outpatient care was already highly rationed, with both primary care and subspecialty clinics closing their doors and all but emergent medical visits discouraged. The RFA sought to explore novel proposals that could address these healthcare gaps. Examples included examination of telemedicine and computer-based approaches to care. The RFA raised the questions of what impact COVID-19 would have on routine childhood primary care, instructions about safety, scheduled immunizations, and access to social services. Of particular interest, applications were sought that addressed the impact of social isolation on childhood mental health.

## Results

### Review Process

Applications were accepted on a rolling basis, with a goal of funding up to nine seed grant projects, for a 1-year period ($10,000 direct costs, no salary support). Contingent upon a successful progress report, a tranche of an additional $5,000 was made available for a second year of funding. In contrast to past NIH-style study section reviews of applications, these applications underwent expedited review by a panel consisting of the corresponding authors of this report, along with three additional community-based investigators with established track records of child health research. If significant concerns were noted by two or more reviewers, applications were not recommended for funding. However, if the application was well-received, an affirmative funding decision was made immediately, typically within 2 weeks of receipt of the application.

### Successful Applications

A total of 11 applications were received and 9 were favorably reviewed and funded. The funded studies were observed to segregate into three general thematic categories (described below; see also Table 2).

**Table 2.** Projects funded by COVID-19 children’s collaborative “rapid response” request for applications (RFA)

Funded applications	
Category	Funded projects
Biology of SARS-CoV-2 infection in children	Neurological manifestations of COVID-19 infection in children Endothelial dysfunction in pediatric patients with COVID-19 infection: identification of biomarkers Antibody epitopes associated with response to spike protein in children with COVID-19: “reverse genetic” analysis of B cell epitopes following pediatric COVID-19 infection
Epidemiology, psychological, familial, and emotional impact of SARS-CoV-2 in children	Evaluation of the prevalence and impact of SARS-CoV-2 infection in children through examination of TriNetX database Sequelae of COVID-19 in children with underlying mental health issues: coping strategies, emotional regulation, and psychological outcomes Examining family coping strategies and potential support mechanisms, including examination of the home food environment (i.e., food insecurity)
Social justice, racism, child abuse in the context of the COVID-19 pandemic	Impact of COVID-19 pandemic on stress physiology Anti-Asian racism targeting youth, impact on mental health, resiliency techniques Impact of COVID-19 on child abuse and neglect

#### Biology of SARS-CoV-2 Infection in Children

Grant 1. “Does Infection with the SARS-CoV-2 Virus Alter Brain Structure and Function?”

*Goal*: To examine by MRI the impact of COVID-19 on brain structure and function, using fMRI, in pediatric patients hospitalized with severe COVID-19 disease.

Grant 2. “Pilot Screening for Urinary Biomarkers of Vasoreactive Endothelial Dysfunction in Pediatric Patients with Suspected COVID-19 Infection.”

*Goal*: To identify biomarkers of severe COVID-19 disease in hospitalized children by measuring urinary metabolites that reflect endothelial cell activation. A secondary aim was to explore potential biomarkers that might correlate with the clinical phenotype of COVID-associated multisystem inflammatory disease of childhood (MIS-C).

Grant 3. “Isolation of Protective Antibodies Against COVID-19.”

*Goal*: Employ reverse genetics of VDJ chain recombination from B-cell populations from children following their convalescence from COVID-19 disease to map diversity and usage of spike protein epitopes corresponding to the humoral immune response.

#### Epidemiology of SARS-CoV-2 Infection in Children

Grant 4. “Epidemiology of Pediatric COVID-19 Infections in the USA.”

*Goal*: To ascertain risk factors for hospital admission of children with COVID-19 infection, using the TriNetX database.

Grant 5. “Emotional and Behavioral Regulation Among Children and Adolescents with Pre-Existing Mental Health Disorders Throughout the COVID-19 Pandemic.”

*Goal*: To explore the impact of pre-existing mental health disorders in children during the COVID-19 pandemic on coping strategies, emotional regulation, and psychological outcomes.

Grant 6. “Working Together, From a Distance: A Qualitative Exploration into How to Best Tailor Our Public Health Response to Support Families During the COVID-19 Pandemic.”

*Goal*: Using existing infrastructure examining family coping and support mechanisms, this project explored how COVID-19 evoked changes to the home food environment and parent feeding practices during the pandemic.

#### Social Justice

Grant 7. “Stress Physiology and Mental Health: Implications for Family Functioning and Child Development During the COVID-19 Pandemic.”

*Goal*: To utilize established tools gauging family stress, such as saliva cortisol levels, in an exploratory study of the impact of the COVID-19 pandemic on stress physiology.

Grant 8. “Anti-Asian Racism During the Coronavirus Pandemic: Youth Risk and Resilience.”

*Goal*: To examine racism targeted at Asian-American youth, measure its impact on mental health, and describe resiliency techniques.

Grant 9. “Impact of COVID-19 on Child Abuse and Neglect in the Twin Cities.”

*Goal*: Describe the patterns of child abuse and neglect emerging during the COVID-19 pandemic and compare to pre-pandemic data.

### Impact

We funded nine applications through this RFA and observed that they fell into three distinct categories (Table 2): the biology of SARS-CoV-2 infection in children; the epidemiology of SARS-CoV-2 infection in children, including impact of pre-existing co-morbidities; and social justice in the setting of the COVID-19 pandemic and the child health implications. We observed that most of these awards represented new, novel collaborations between our two institutions. The relatively distinct nature of these categories facilitated the presentation of the research at three separate “Child Health Research Forum” workshops on 5/12/2021, 6/9/2021, and 6/30/2021. Of the nine applications, all but one group applied for and received a second tranche of funding, through a supplemental RFA in year 2 of the program, for continuation of the research collaboration. Part of the success of these programs was based upon the ability of the investigative teams to identify research topics and questions that were of interest to the participants [8]. To date, investigators supported by the COVID-19 “rapid response” RFA have published five papers related to research topics outlined in these awards [9–13], and two groups have leveraged this support to successfully acquire additional extramural funding.

## Discussion

An important goal of the CTSA funding mechanism is to engender and nurture robust community-university collaborations that advance implemenation of translational research findings into clinical practice. One approach to foster collaboration is through creation of community advisory boards (CABs). Most (89%) CTSA awardees report having a CAB [14]. Collaboration between academic researchers and community members, in particular clinicians and care organizations, is a critical programmatic component of community-engaged health research, and a CAB can play an important role in identifying high-priority areas for future work [15,16]. However, even with active CABs, there are important gaps in study participation in clinical trials, particularly in special populations such as elderly patients, rural communities, historically under-represented minorities, and children [8]. The under-funding of child health-related research, particularly when calculated by resources invested by the NIH as a function of the relative population distribution of children in the USA, has been noted in previous studies [17–19]. These gaps have only been exacerbated by the COVID-19 pandemic [2–4].

To address these unmet needs for advocacy specifically centered on child health research supported by the CTSA mechanism, and in recognition of the value that pediatric collaborative networks can play in enhancing child health research [20–23], Children’s MN partnered with the UMN CTSI (in 2010) to form a Child Health Advisory Board. Members include investigators engaged in child health research from various UMN AHC colleges and programs; investigators from Children’s MN; and a representative from the Department of Pediatrics at HCMC. The Advisory Board meets 2–4 times annually to discuss programmatic collaborative opportunities, to organize seminars and colloquia, and to administer grant programs.

In the setting of the COVID-19 pandemic, a change in grant administration was undertaken, namely to replace the NIH study section-style award mechanism with a series of “rapid response” grants. The upheaval engendered by the COVID-19 pandemic engendered a need to pivot toward projects that helped to enhance an improved understanding of COVID-19’s impact as a virus and pandemic, particularly as it related to child health. Grants were awarded on a “rolling” basis following a rapid review by senior staff at our respective institutions and community reviewers. Although the content request was not restrictive, the RFA requested applications in three key areas of interest (Table 1) focused on: the impact of COVID on families; the biology of SARS-CoV-2 virus as it related to children; and the impact of the COVID-19 pandemic on pediatric clinical practice.

We found that the transition from the pre-existing funding mechanism to the “rapid response” approach that was implemented during the pandemic was straightforward and well-received by both institutions. We attributed this to several factors: 1) close personal working relationships between the co-PIs of the respective institution; 2) a small but committed working group of Child Health Advisory Committee members who were available on short notice for e-mail correspondence, Zoom meetings, and conference calls; and 3) a paucity of administrative overlay. In general, we found that the program made the benefits of a highly functioning academic-community child health partnership very clear, and we propose this “rapid response/rapid review” model as a paradigm for future collaborative funding initiatives. Although events as dramatic as an infectious disease pandemic are rare and difficult to anticipate, there is nonetheless great value in generating a more nimble, timely response to public health challenges. We observed that our previous collaborative funding opportunities, although successful, were patterned upon the experience that many investigators are familiar with – namely, an NIH-style model in which applications are reviewed by study sections, followed by second-level administrative and budgetary review. Such processes can take months to bring to fruition. Given the urgency of the COVID-19 pandemic, such lengthy delays were believed to be unacceptable. As a testament to the enthusiasm of the sponsoring institutions, both the UMN and Children’s MN have agreed to continue to fund this collaborative grant program into the future.

### Limitations

In this communication, we share our institution’s experience with a “rapid response” funding program for COVID-19 applications germane to child health-specific research related to the SARS-CoV-2 virus, building upon an established infrastructure of collaboration between our institutions and utilizing the expertise and leaderhip of an existing Child Health Advisory Board. Modification of our pre-existing grant program led to an increased number of funded awards. One limitation is that we cannot gauge whether the award of multiple, smaller seed grants had a greater overall impact on the generation of new knowledge about the child health manifestations of COVID-19 than might have been realized by a single, larger grant. On the other hand, a seed grant model may have encouraged more risk taking and consideration of more novel projects. We do note that most partnerships funded by this award are still intact as of this writing, and most (8/9) awardees applied for a second year of funding to continue to support their collaborations. Thus, we think this program was successful in creating new research partnerships between our two institutions. Like many other healthcare institutions, our institutions were impacted by the departures of long-term research faculty and staff (including some members of these nine research teams) during the pandemic who sought new employment opportunities with other employers [24]. We noted that among research team members who left for other opportunities, many nonetheless continued to remain engaged and involved in the nine funded research projects, even in their new roles at other institutions, suggesting their strong commitment and interest in the program, in their collaborations, and in the study of COVID-19 infection in children.

Another limitation of our rapid response program was the administrative delay in regulatory and grants management processes, including budgetary processes. A pre-requisite for the program was that the principal co-PIs had to have primary appointments at Children’s MN or the UMN AHC, respectively. Coordination across the two institutions created challenges, as did the timing of study approval for our two respective IRBs. Looking to the future, we plan to address this issue by exploring a joint IRB mechanism between the two institutions to help accelerate the pace and timing of future projects. An example of such a strategy is the Trial Innovation Network (TIN), which represents a collaborative initiative with the CTSA Program and other NIH Institutes that attempts to address roadblocks to collaborative research [25]. We are hopeful as the UMN and Children’s MN collaboration continues and grows that strategies articulated by the TIN, including single-IRB submissions, can help improve the timeliness of project initiation.

In summary, the rapid response COVID-19 seed grant program was well received. Most research teams were comprised of novel working partnerships, fulfilling a key goal of the RFA. Regulatory and grants management processes encountered set-backs but were manageable. Although COVID-19 lockdowns made clinical research enrollment challenging, adaptations at both institutions, including remote consent procedures and increased use of E-visit encounters, helped ensure the feasibility of study enrollment. Most research teams applied for, and received, a second year of funding, and research continues. Research partnerships resulted in three well-attended symposia, as well as several peer-reviewed publications and extramural grant awards. Transmission of SARS-CoV-2 among children has emerged as a driving force that currently sustains the COVID-19 pandemic [26]. Moreover, the health consequences of COVID-19 in children, including the mental health impact of mitigation measures, are distinct from those in adults. Until vaccines are more broadly implemented for infants and young children, a nimble response to COVID-19 is required. CTSA-funded community-based partnerships are very well-described [27,28], including community engagement programmatic responses with metrics generated by the COVID-19 pandemic [29,30], but this report highlights one of the few community engagement responses to the pandemic that is uniquely child health oriented. An increased focus on the importance of community-based partnerships that are uniquely concerned with child health issues is an important goal for future collaborations and will help address the under-representation of children in clinical research as we continue to confront these challenges in the post-pandemic world [31].
